# The influence of palm fiber reinforcement on the cement content of vegetated concrete substrate under the condition of equal strength

**DOI:** 10.1371/journal.pone.0311928

**Published:** 2024-10-17

**Authors:** Xiaole Huang, Xue An, Gang Zeng, Shiyuan Xiong, Xiaojun Sun

**Affiliations:** 1 School of Architectural Engineering, Xiangyang Vocational and Technical College, Xiangyang, China; 2 School of Civil Engineering and Architecture, Hubei University of Arts and Science, Xiangyang, China; 3 School of Chemical & Environmental Engineering, Pingdingshan University, Pingdingshan, China; Covenant University, NIGERIA

## Abstract

Vegetated concrete substrate (VCS) is a kind of ecological cemented soil, which has very wide application prospect in high and steep rock slope eco-protection. Cement is an important component of VCS, but it has high energy consumption and environmental pollution. Fiber reinforcement plays an positive role in improving the mechanical properties of soil, and its use as a substitute for cement content in VCS under the condition of equal strength is rarely investigated. In this study, the unconsolidated-undrained (UU) triaxial compression test of unreinforced substrate as blank control samples (BCS) and reinforced substrate as fiber reinforced samples (FRS) were carried out. The test results showed that the stress-strain curve of VCS can be divided into compaction stage, elastic stage, plastic stage and strain hardening stage. The average peak strength increased by 34.3kPa, 53.6kPa, 218kPa and 81.8kPa as cement content of VCS was 0%,4%, 6% and 8%, respectively. The relationship between the peak strength and cement content of VCS could be better fit by Boltzmann function. The mathematical model of fiber instead of cement in VCS under the condition of equal strength was established. It is found that there is a critical point of cement content according to the mathematical model. The cement of VCS can be completely replaced by plam fiber as the cement content is less than the critical point. While the cement content is higher than the critical point, the cement of VCS can be partially replaced by plam fiber. The decrease of average cement content was 17.23%, 19.00%, 24.27% and 25.34% with 0.2%, 0.4%, 0.6% and 0.8% fiber content in reinforced substrate, respectively. The theoretical method and interpolation method for fiber substitute cement content of VCS under equal strength condition were proposed, which can provide technical guidance for ecological slope protection engineering practice of vegetated concrete.

## 1 Introduction

Earthwork excavation is very common in transportation, water conservancy, mining and other engineering practices, which will not only destroy the ecological environment, but also form a large number of exposed slopes [1–3]. These ecological wounds are difficult to recover under natural conditions, and there are geological hazards such as landslides and collapses [4,5]. Vegetation concrete slope protection technology is widely used in ecological restoration of exposed slope [6–8]. The technology is to spray the mixture of planting substrate and planting green seeds onto the slope surface to form a shallow stable layer of slope with a thickness of about 10cm, which has good ecological and landscape effects [9–11].

VCS is the core of ecological slope protection technology of vegetated concrete [12]. VCS is a mixture of cement, soil, organic matter, ecological amendments and water in a certain proportion [13]. The substrate must not only have conditions that facilitate for plant growth, but also need to have appropriate strength [14]. The cohesiveness and strength of VCS are formed by the hydration reaction of cement. However, cement has a certain alkaline stress effect on plant growth. The higher the cement content, the greater the stress effect of cement on plants [8]. The reduction of cement content in the substrate is beneficial to plant growth and slope ecological restoration from an ecological point of view [13]. In addition, the cement industry has become a major emitter of carbon dioxide due to its inherent raw material structure and production process limitations [15,16]. Each 1t of cement clinker produced will emit 0.894 ~ 1.215t CO_2_.At present, the cement industry accounts for about 13.5% of the country’s total carbon emissions in China. As one of the world’s largest cement producers and consumers, the cement industry has become a key area for China to achieve the goal of "carbon peak and carbon neutrality".

The reduction of the amount of cement in the substrate has great economic and environmental benefits. However, the strength of the substrate also decreases with the reduction of the cement content, which will affect the stability of the substrate on the slope. Therefore, it is particularly necessary to reduce the amount of cement by adding appropriate reinforcement materials under the premise that the strength of the substrate meets the requirements. Plam fiber can not only improve strength and other engineering mechanical properties of the substrate, but also contribute to the recycling of waste resources [17–22]. Fiber can improve the strength and durability of concrete [23–25], but there are few reports on the content of fiber instead of cement.Therefore, palm fiber is selected as the reinforced material of the substrate in this study, and the strength of 4 unreinforced substrate samples and 16 reinforced substrate samples are determined by unconsolidated undrained triaxial shear test. The substitution effect of plam fiber on cement content in VCS is studied, and the method of substituting plam fiber for cement is put forward based on the condition of equal strength.

## 2 Material and methods

### 2.1 Materials

The VCS is composed of soil, organic matter, cement and habitat modifying agent in this study. The soil is collected from a farmland in Yichang of Hubei Province, China. The organic matter is pine sawdust purchased in the market. The particle percentage of soil and sawdust is shown in Table 1. It can be seen from Table 1 that the soil texture is silty sand (SM) containing fine grained soil. The cement is P·C 32.5 composite Portland cement, and its physical and mechanical indexes are shown in Table 2. The function of habitat modifying agent is to activate the substrate to facilitate plant growth, and the detailed information can visit the official website of Hubei Runzhi Ecological Technology Co., LTD (www.runzhist.com).

**Table 1:** Particle percentages of the soil and pine sawdust.

Particle size /mm	≥2	2~1	1~0.5	0.5~0.25	0.25~0.1	0.1~0.074	<0.074
Soil	0	21.34	25.46	19.88	16.88	8.17	8.27
Pine sawdust	12.06	12.06	40.75	24.76	7.01	3.34	0

**Table 2:** The basic parameters of cement.

Cement	SO_3_ (%)	MgO (%)	Cl^-^ (%)	Initial setting time(min)	Final setting time(min)	Rupture strength (MPa)		Compressive strength (MPa)	
						3d	28d	3d	28d
P.C 32.5	≤3.5	≤6.0	≤0.06	≥45	≤600	≥2.5	≥5.5	≥10	≥32.5

The reinforced material is plam fiber in this study. The physical and mechanical indexes of plam fiber are shown in Table 3. As can be seen from Table 3, plam fiber is a kind of medium strength and high elongation fiber.

**Table 3:** Physical and mechanical parameters of palm fiber.

Diameter /μm	Length /cm	Elasticity modulus /GPa	Tensile strength /MPa	Elongation ratio /%	Acid and alkali resistance
199~539	2.0	0.44~1.09	89~222	14.68~23.45	Strong

### 2.2 Sample preparation

#### 2.2.1 Test scheme

The weight of each component in VCS is usually determined by the dry weight of soil in the practice of ecological slope protection engineering. The content of sawdust and ecological amendments is set to a fixed value, 6% and 4% of the dry soil mass respectively. The cement content is set at 0%, 4%, 6% and 8% of the dry soil mass according to the references. The plam fiber content is based on the dry weight of mixtures such as soil, sawdust, cement and ecological amendments in this study. The content of plam fiber shall not exceed 0.8% of the dry weight of the mixture according to the pre-mix test. Therefore, the plam fiber content is set to four levels, 0.2%, 0.4%, 0.6% and 0.8% of the dry weight of the mixture. The BCS is with no addition of fibers and contains 0%, 4%, 6% and 8% cement. The FRS is manufactured by adding plam fiber into VCS in the proportion of 0.2%, 0.4%, 0.6% and 0.8% respectively. The test scheme of this study is shown in Table 4.

**Table 4:** The test scheme of this study.

Group	Composition of the substrate sample								
	Content of each component of the substrate (%)				Fiber content (W_f_ /%)				
	Soil	Cement	Pine sawdust	Habitat modifying agent	BCS	FRS			
Ⅰ	100	0	6	4	0.0	0.2	0.4	0.6	0.8
Ⅱ	100	4			0.0	0.2	0.4	0.6	0.8
Ⅲ	100	6			0.0	0.2	0.4	0.6	0.8
Ⅳ	100	8			0.0	0.2	0.4	0.6	0.8

**Fig 1:**
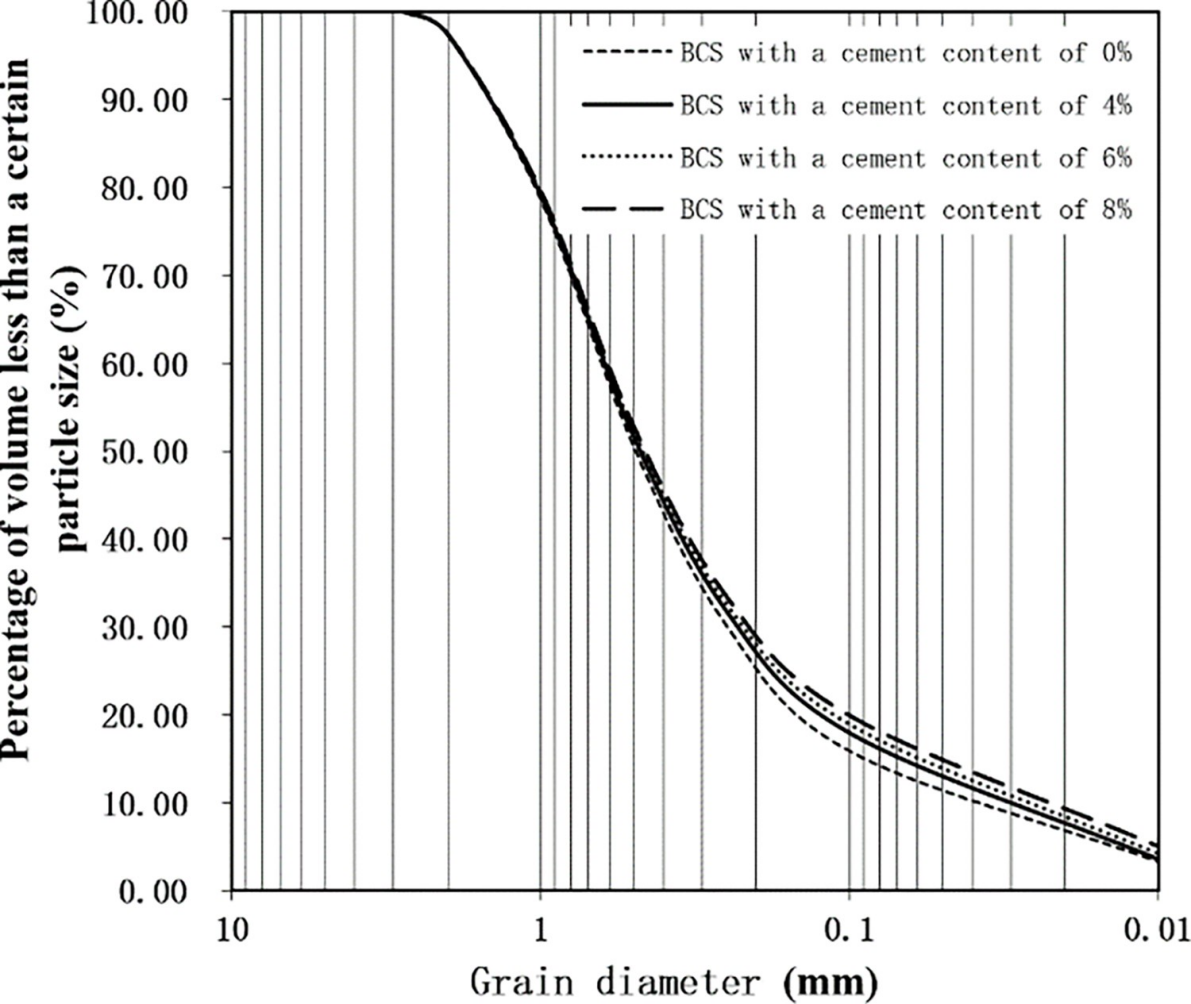
Particle size distribution curves of BCS.

The particle size distribution curves of the BCS is showed in Fig 1. It can be seen from Fig 1 that the particle size distribution range of BCS is wide and the curve is relatively gentle.

The characteristic particle sizes *d*_*60*_、*d*_*30*_、*d*_*10*_ as well as their inhomogeneity coefficient *C*_*u*_ and curvature coefficient *C*_*c*_ of BCS can be obtained from Fig 1, and the results are shown in Table 5. As can be seen from Table 5, *C*_*u*_ is greater than 10 and *C*_*c*_ is in the range of 1–3, which indicates that the BCS belong to well-graded soil.

**Table 5:** Characteristic particle sizes, nhomogeneity coefficient and curvature coefficient of BCS.

Cement content/%	*d*_*60*_	*d*_*30*_	*d*_*10*_	*C*_*u*_	*C*_*c*_
0	0.634	0.24	0.04	15.85	2.27
4	0.63	0.225	0.030	21.00	2.68
6	0.626	0.221	0.028	22.36	2.79
8	0.621	0.220	0.023	27.00	2.80

#### 2.2.2 Sample preparation

**Fig 2:**
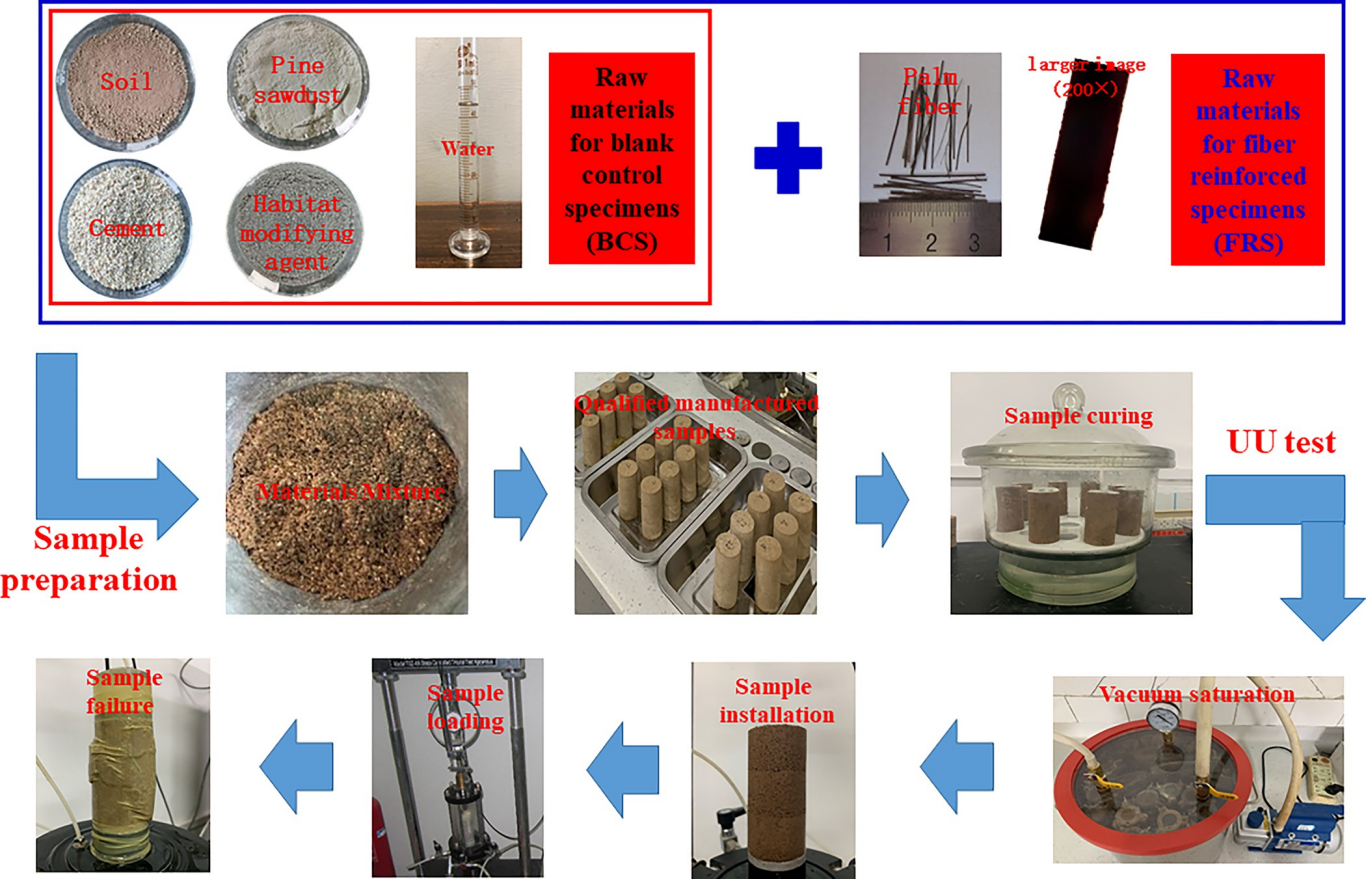
The process of sample preparation.

The soil is naturally air-dried, ground and sieved by 2mm [26]. The sawdust should be air-dried and screened by 2mm before sample preparation. The plam fiber is cut and broken to less than 2mm.The specimen size is a cylinder with diameter of 39.1mm and height of 80mm. The initial moisture content and dry density of VCS are determined by engineering experience to be 20% and 1.35g/cm^3^, respectively [13]. Each component of VCS is initially weighed (with an accuracy of 0.01 g) according to the requirements and mixed together, then the water required is added and mixed with the dry mixture. The watered mixture is stored in an airtight container for 30 min, so that the water is fully and evenly absorbed by all components. The mixed material is evenly pressed into the cylinder sampler in 3 times one by one. The quality inspection is carried out on the sample after compaction is completed, and samples with a quality error of less than ± 1% are considered qualified [13]. The weight of each sample after pressed in cylinder mould is about 158.5g. The prepared samples are cured in a moist chamber for 7 days before they are subjected to subsequent testing. The process of sample preparation is showed in Fig 2.

### 2.3 Method

#### 2.3.1 Test method

The consolidation and drainage effects of the VCS on the side slope are not obvious, which is mainly due to its thin thickness. Therefore, the unconsolidated undrained (UU) triaxial test method is used to measure the strength of the VCS. Strain controlled triaxial shear instrument is used in the test equipment, which can automatically record the deviational stress and axial load of the sample. The confining pressure is set at 50kPa and the shear rate is 0.9 mm/min. The failure criterion of the sample is the peak point of deviational stress. If there is no peak point, the peak strength is the deviational stress as the axial strain reaches 15% [13].

#### 2.3.2 Analytical method

The effect of fiber reinforcement on the strength of the substrate is analyzed according to the UU triaxial test results, and the functional relationship between the strength of the blank substrate and the strength of the reinforced substrate and the cement content Is established respectively. Based on the principle of equal strength, the substitution effect of fiber on cement content of substrate Is analyzed under the premise of equal strength.

## 3 Results and analysis

### 3.1 Stress-strain curves of VCS

**Fig 3:**
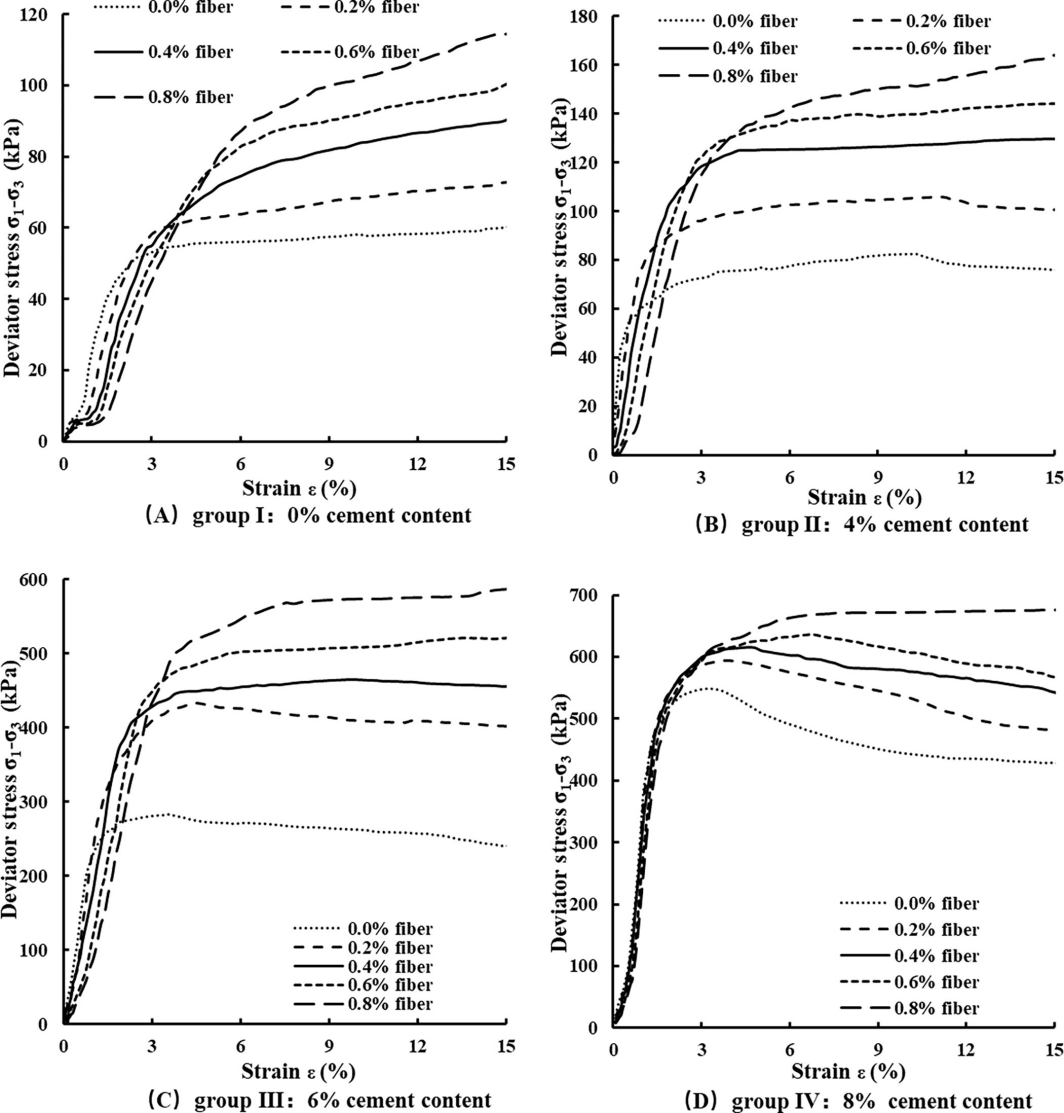
Stress-strain curves of VCS samples (A) Group I (B) GroupⅡ (C) Group Ⅲ (D) GroupⅣ.

The stress-strain curves of the four groups are shown in Fig 3. In Fig 3, the specimens with a fiber content of 0.0% are identified as BCS (blank control samples), while the specimens with fiber contents of 0.2%, 0.4%, 0.6%, and 0.8% are designated as FRS (fiber reinforced samples).

As can be seen from Fig 3, the stress-strain curves of the four groups of specimens are basically the same, which can be characterized of four typical phases, namely initial deformation, elastic deformation, yielding, and hardening stage or softening stage. The compaction characteristics of the specimens in group Ⅰ are the most obvious and the compaction feature become more and more obvious as the fiber content increases. In addition, the deviational stress of the BCS increased with the increase of cement content and their brittleness became obviously enhanced simultaneously. The FRS hold the increasing stresses with fiber contents and their brittle characteristics were significantly improved at the same time. In the same group, the BCS has the most obvious brittle characteristics and the brittleness of the FRS are gradually weakened with the increase of fiber content. The stress-strain curves of group Ⅰ, Ⅱ, Ⅲ and Ⅳ are characterized as strain hardening type, weak strain hardening type, ideal elastic-plastic strain and strain softening strain, respectively.

### 3.2 Ultimate strength of VCS

**Fig 4:**
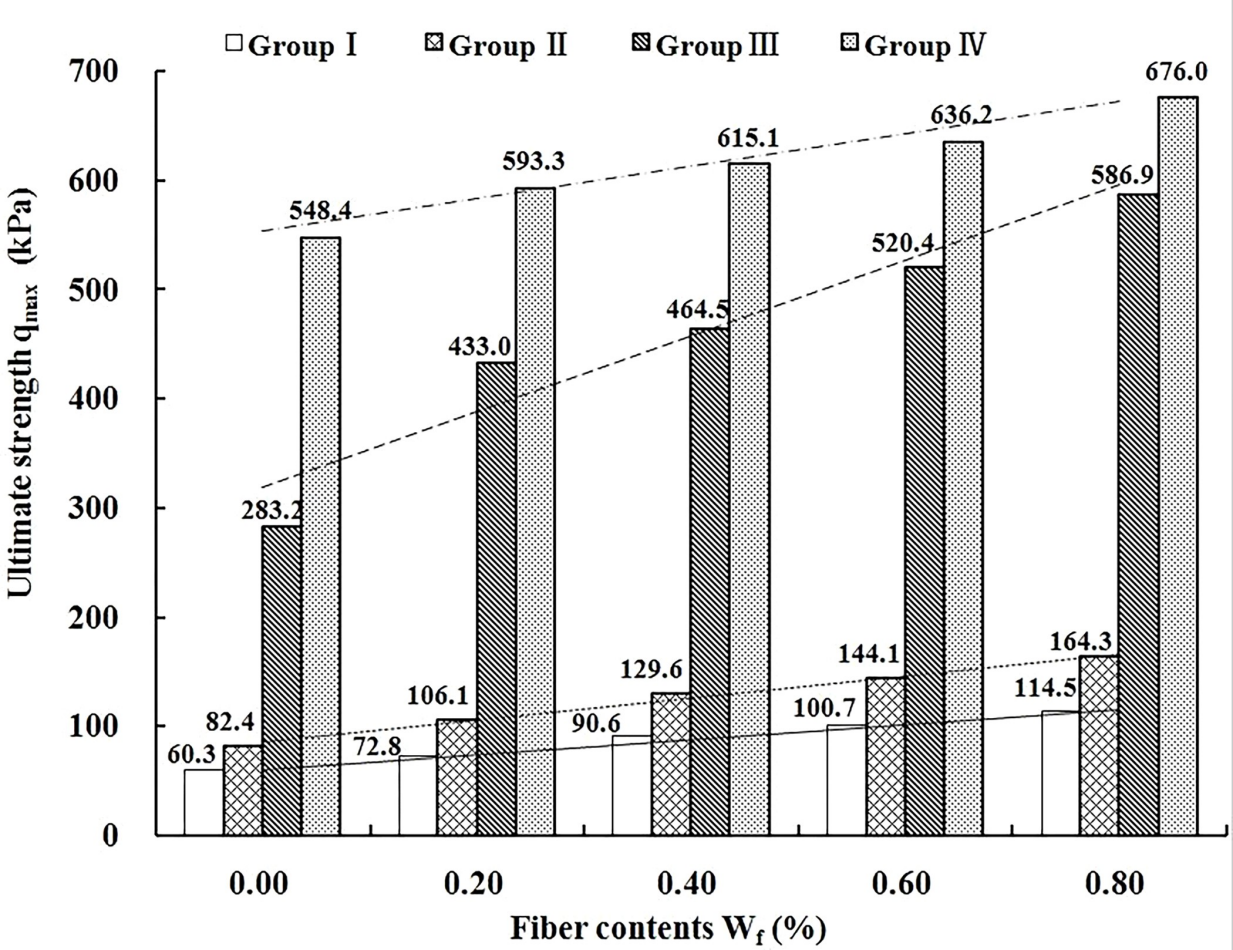
Relationship between ultimate strength and fiber contents of VCS.

The results of the ultimate strength of the four groups of specimens are shown in Fig 4.

As can be seen in Fig 4, compared with group Ⅰ, the ultimate strength of the BCS in group Ⅱ did not increase significantly while the ultimate strength of Ⅲ and Ⅳ increase by 3.7 times and 8.1 times, respectively. The trend of the ultimate strength of the FRS is consistent with that of the BCS. Compared to the BCS (sample with a fiber content of 0.0%), the average increases in ultimate strength for the FRS (samples with fiber contents of 0.2%, 0.4%, 0.6%, and 0.8%) across the four groups are 34.3 kPa, 53.6 kPa, 218 kPa, and 81.8 kPa, respectively. The average increase of the ultimate strength first increased and then decreased with the increase of cement content and the values were 57%, 65%, 77% and 15%, respectively. When the cement content is 6%, the FRS has the largest increase of the ultimate strength. This is because that the strengths of VCS are affected by the coupling actions of fiber and cement. When the cement content is zero or low (groups Ⅰ and Ⅱ), the reinforcement potential of the fiber is not fully developed because the substrates would be weakly cemented in term of the relatively low hydrate products generated. As the substrates become increasingly improved (groups Ⅲ), the optimum potential would be achieved when the load-bearing capacity of substrate is compatible to that of fiber. While when the cement content is high (group Ⅳ), the capacity of the substrates becomes far higher than the potential limit of fiber, the potential of fiber would be partially inhibited and incompletely achieved [13].

### 3.3 Relationships between ultimate strength and cement content of VCS

Based on the results of the four groups of specimens’ ultimate strength, the linear function (LF), the exponential function (EF) and the Boltzmann function (BF) under the growth/sigmoidal option of the Origin 7.0 software were used to fit the experimental data for math model. The fitting results are shown in Table 6. The expression of BF is shown in Eq 1.


$$q_{\max}=\frac{a_{1}-a_{2}}{1+e^{\frac{X_{c}-x_{0}}{d}}}+a_{2}$$ (1)

**Table 6:** The parameters and correlation coefficients of master curves.

*W*_*f*_/%	Parameters of BF				LF	EF	Correlation coefficients		
	*a*_*1*_	*a*_*2*_	*x*_*0*_	*d*			BF	LF	EF
0.0	60.3	548.4	6.1595	0.4	*q*_*max*_ = 58.047*X*_*c*_-17.637	*q*_*max*_ = 46.727*e*^0.2826*Xc*^	0.993	0.766	0.866
0.2	72.8	593.3	5.3885	0.4	*q*_*max*_ = 67.011*X*_*c*_-0.2514	*q*_*max*_ = 59.644*e*^0.2808*Xc*^	0.975	0.815	0.865
0.4	90.6	615.1	5.33323	0.4	*q*_*max*_ = 67.917*X*_*c*_+19.323	*q*_*max*_ = 75.891e^0.2565*Xc*^	0.975	0.821	0.869
0.6	100.7	636.2	5.1924	0.4	*q*_*max*_ = 70.917*X*_*c*_+31.223	*q*_*max*_ = 85.623*e*^0.2496*Xc*^	0.986	0.817	0.862
0.8	114.5	676.0	5.093	0.4	q_max_ = 75.684X_c_+44.846	q_max_ = 98.725e^0.2424Xc^	0.994	0.811	0.858

In Eq (1), *q*_*max*_ is the ultimate strength of the specimen and *X*_*c*_ is the cement content. The characters including *a*_*1*_, *a*_*2*_, *x*_*0*_ and *d* are the regression coefficients of the fitting function, where *x*_*0*_ is the inflection point, *q*_*max*_ *= a*_*1*_ and *q*_*max*_ *= a*_*2*_ are the two asymptotes of the fitting curve.

As can be seen from Table 6, the correlation coefficients of LF and EF ranged from 0.766 to 0.821 and 0.858 to 0.869, with average values of 0.806 and 0.864, respectively. In contrast, the range of correlation coefficient of BF (0.975–0.994) and its average value (0.985) are much higher than those of LF and EF, which means that the BF can better reflect the relationship between the ultimate strength and cement content of the VCS.

**Fig 5:**
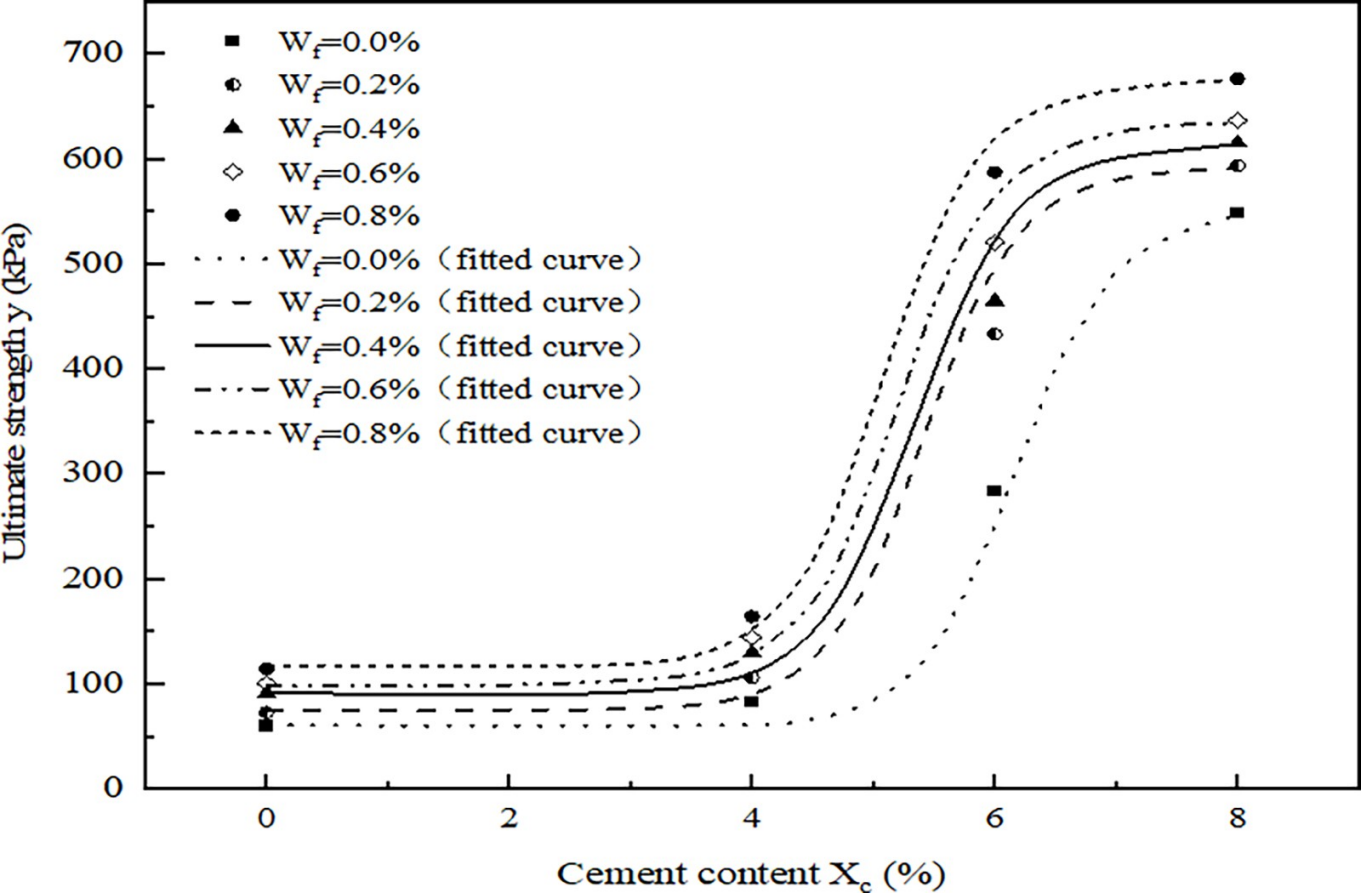
Fitting relationships between the ultimate strength and cement content of VCS.

Fig 5 shows the relationship between the ultimate strength and cement content of the VCS fitted by BF.

It can be seen from Fig 5 that the fitting curves of BF are S-shaped. When the cement content is less than 4%, the ultimate strengths of VCS increase slowly with the increase of cement content. When the cement content ranges from 4% to 6%, the ultimate strength of VCS enters a rapid growth stage. However, with a further increase in cement content (more than 6%), the growth rate of the ultimate strength has the trend of decreasing.

## 4 Effect of palm fiber as a substitute for cement in VCS under the condition of equal strength

**Fig 6:**
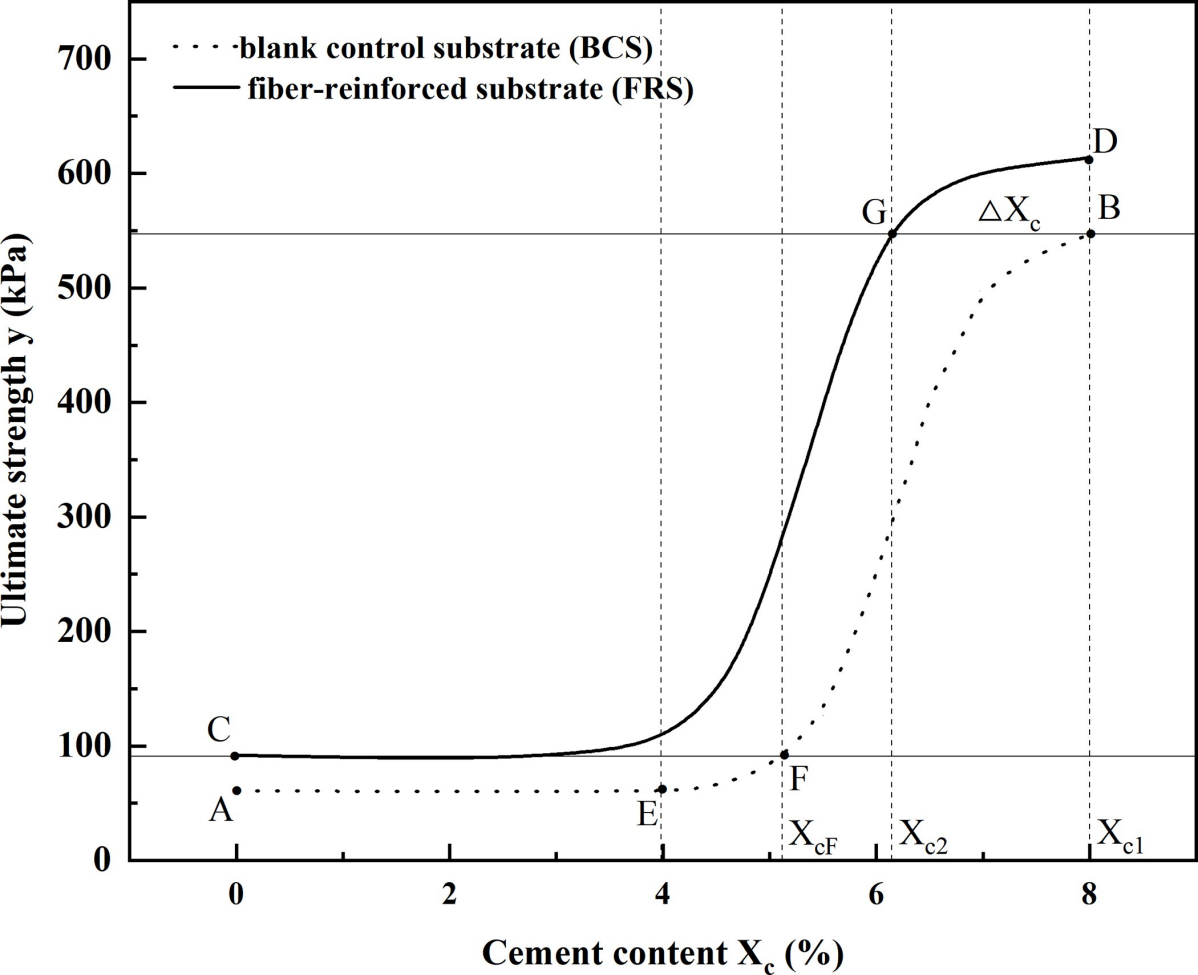
Diagram of cement content of the relationship between BCS and FRS.

In this research, the equal-strength function relationships between the VCS and the FRS are established on the assumption that the self-stability of the FRS could meet the engineering requirements as long as the FRS has the same strength with the BCS (Fig 6).

In Fig 6, A and C are the points on AB and CD curves where the cement content is 0%, respectively. B and D are the points on AB and CD curves where the cement content is 8%, respectively. E is the point at 4% cement content on AB curve. G is the intersection point of the line parallel to the horizontal axis and the CD curve. F is the intersection point of the line parallel to the horizontal axis and the AB curve. F is the critical point on AB curve and the corresponding cement content is recorded as *X*_*c(F)*_.

Because the cement content of VCS in engineering practice ranges from 4% to 8%, in this study only the equal strength equations in the segment of EB curve of the BCS were established. The EB curve is divided into EF segment and FB segment with F point as the boundary. And the strength corresponding to the EF curve are lower than that of the C point (*Xc* = 0%, *W*_*f*_ = 0.2%,0.4%,0.6%,0.8%) on FRS curve. Combined with the variation principle of the ultimate strength of BCS, the strength of FRS can be equal to that of the BCS by keeping the cement content constant (*Xc* = 0%) and reducing the fiber content appropriately. In contrast, there is an equal strength on CD curve of the FRS to every strength on FB curve of the BCS, which means that the equal strength model of the BCS and the FRS can be established directly through the Boltzmann functions of them. The methods and steps about how to construct the iso-strength functional relationship between the BCS and the FRS for the EF and FB sections are as follows.

Using the strength fitting curve (AB) of the BCS as standard, and taking the fitting curve (CD) of the FRS with a fiber content of 0.4% as an example, the establishment process of the equal strength function relationships are as follows.

### 4.1 The equal strength functional relationship between the BCS of EF segment and the FRS

The equal strength functional relationship between the BCS of the EF segment and the FRS is established as follows.

Step 1: Using linear function to fit the relationship between the ultimate strength *q*_*max(1)*_ and the fiber content of the FRS (Group I, Fig 4). The result is shown in Formula (2).

Step 2: Substitute the master curve parameters of the BCS in Table 6 into Formula (1) to obtain the functional relationship between the ultimate strength *q*_*max (0)*_ and the cement content *X*_*c(0)*_ of the BCS. The fitting result is shown in Formula (3).

Step 3: Unit Formula (2) and Formula (3) under the condition of equal strength for the BCS and the FRS (*q*_*(max(1)*_ *= q*_*(max(0)*_), and the relationship between the cement content *X*_*c(0)*_ of the BCS and the fiber content *W*_*f*_ of the FRS is shown in Formula (4).


$$q_{\max(1)}=68.15\times W_{f}+60.52,R^{2}=0.9943$$ (2)

$$q_{\max(0)}=584.4-\frac{488.1}{1+e^{\frac{X_{c(0)}-6.1595}{0.4}}},(4\leq X_{c(0)}<X_{c(F)})$$ (3)

$$W_{f}=\frac{487.88-488.1/(1+e^{\frac{X_{c(0)}-6.1595}{0.4}})}{68.15}$$ (4)

It can be seen from the Formula (4) that under the condition of equal strength, when the cement content of the VCS is low, the cement can be completely replaced by palm fibers.

Table 7 shows the one-to-one relationship between cement content *X*_*c(0)*_ of the BCS and fiber content *W*_*f*_ of the FRS for some points on EF curve under the condition of equal strength, which can be used as a reference for engineering practice.

**Table 7:** The one-to-one relationship between the X_c(0)_ of the BCS and the W_f_ of the FRS on EF curve.

Cement Content of the BCS *X*_*c(0)*_ /%	4.0	4.2	4.4	4.6	4.8	5.0	5.2
Fiber content of the FRS *W*_*f*_ /%	0.03	0.05	0.08	0.14	0.23	0.37	0.59

### 4.2 The equal strength functional relationship between the BCS of FB segment and the FRS

The specific steps for establishing the equal strength relationship between the BCS of the FB section and the FRS are as follows.

Step 1: Substitute the master curve parameters of the BCS in Table 6 into Formula (1) to obtain the functional relationship between the ultimate strength *q*_*max (0)*_ of the BCS and the cement content *X*_*c(0)*_. The result is shown in Formula (5).

Step 2: Substitute the master curve parameters of the FRS in Table 6 into the Formula (1) to obtain the functional relationship between the ultimate strength *q*_*max(2)*_ of the FRS and the cement content *X*_*c(2)*_. The result is shown in the Formula (6).

Step 3: The equal strength model of the BCS of the FB section and the FRS can be obtained by uniting the Formulas (5) and (6) (*q*_*max(0)*_ = *q*_*max(2)*_), and the results is shown in the Formula (7).


$$q_{\max(0)}=584.4-\frac{488.1}{1+e^{\frac{X_{c(0)}-6.1595}{0.4}}},(X_{c(F)}\leq X_{c(0)}\leq 8)$$ (5)

$$q_{\max(2)}=\frac{a_{1}-a_{2}}{1+e^{\frac{X_{c(2)}-x_{0}}{d}}}+a_{2}$$ (6)

$$X_{c(2)}=d\times \ln\frac{a_{1}-60.3+(a_{1}-584.4)\times e^{\frac{X_{c(0)}-6.1595}{0.4}}}{60.3-a_{2}+(584.4-a_{2})\times e^{\frac{X_{c(0)}-6.1595}{0.4}}}+x_{0}$$ (7)

In Formula (7), the values of *a*_*1*_, *a*_*2*_ and *x*_*0*_ can be scraped from Table 6. As we can see from Fig 6 that when the cement content in the BCS ranges from *X*_*c(F*)_ to 8 (FB segment), the cement in BCS can be partially replaced by the palm fiber under the condition of equal strength, and Formula (7) gives the corresponding relationship between the cement content *X*_*c(0)*_ of the BCS and the cement content *X*_*c(2)*_ of the FRS.

### 4.3 Effect of palm fiber reinforcement on cement content for VCS under the condition of equal strength

It can be seen from the above analysis that when the cement content in BCS is low (EF section on AB curve), the cement in BCS can be completely replaced by palm fiber with a stunning reduction of 100%. When the cement content in BCS is high (FB section on AB curve), the cement content in BCS can be partially replaced by palm fiber. Under the condition of equal strength, the reduction quantity of the cement content of the BCS could be calculated by the Formula (8).


$$\Delta X_{c(0)}=X_{c(0)}‐X_{c(2)}$$ (8)

Table 8 shows the cement content *X*_*c(0)*_ of the BCS, the cement content *X*_*c(2)*_ of the FRS, the reduction of cement content *△X*_*c*_ and the reduction margin of cement content *△X*_*c*_*/X*_*c(0)*_ under the condition of equal strength.

**Table 8:** Comparison Chart of *X*_*c(0)*_, *X*_*c(2)*_, *△X*_*c*_ and *△X*_*c*_*/X*_*c(0)*_.

*X*_*c(0)*_/%	*W*_*f*_ = 0.2%			*W*_*f*_ = 0.4%			*W*_*f*_ = 0.6%			*W*_*f*_ = 0.8%		
	*X*_*c(2)*_	*△X*_*c*_	*△X*_*c*_ */X*_*c(0)*_	*X*_*c(2)*_	*△X*_*c*_	*△X*_*c*_ */X*_*c(0)*_	*X*_*c(2)*_	*△X*_*c*_	*△X*_*c*_ */X*_*c(0)*_	*X*_*c(2)*_	*△X*_*c*_	*△X*_*c*_ */X*_*c(0)*_
4.8	3.36	1.44	29.91%									
5.0	3.92	1.08	21.56%									
5.2	4.24	0.96	18.39%	3.77	1.43	27.49%	2.11	3.09	59.35%			
5.4	4.50	0.90	16.64%	4.26	1.14	21.18%	3.95	1.45	26.78%	3.46	1.94	35.88%
5.6	4.73	0.87	15.53%	4.56	1.04	18.57%	4.34	1.26	22.58%	4.09	1.51	26.94%
5.8	4.94	0.86	14.77%	4.81	0.99	17.12%	4.61	1.19	20.55%	4.41	1.39	23.89%
6.0	5.15	0.85	14.24%	5.02	0.98	16.26%	4.84	1.16	19.41%	4.66	1.34	22.35%
6.2	5.34	0.86	13.90%	5.22	0.98	15.77%	5.04	1.16	18.78%	4.86	1.34	21.56%
6.4	5.52	0.88	13.74%	5.40	1.00	15.58%	5.21	1.19	18.54%	5.04	1.36	21.30%
6.6	5.69	0.91	13.79%	5.56	1.04	15.69%	5.37	1.23	18.65%	5.18	1.42	21.47%
6.8	5.84	0.96	14.09%	5.70	1.10	16.11%	5.50	1.30	19.12%	5.30	1.50	22.05%
7.0	5.97	1.03	14.68%	5.82	1.18	16.86%	5.60	1.40	19.94%	5.39	1.61	22.99%
7.2	6.08	1.12	15.57%	5.91	1.29	17.92%	5.68	1.52	21.07%	5.46	1.74	24.22%
7.4	6.16	1.24	16.74%	5.98	1.42	19.25%	5.74	1.66	22.45%	5.50	1.90	25.65%
7.6	6.22	1.38	18.16%	6.02	1.58	20.77%	5.78	1.82	23.98%	5.53	2.07	27.21%
7.8	6.26	1.54	19.74%	6.05	1.75	22.41%	5.80	2.00	25.61%	5.55	2.25	28.83%
8.0	6.29	1.71	21.41%	6.07	1.93	24.11%	5.82	2.18	27.27%	5.56	2.44	30.46%
Average value		1.09	17.23%		1.26	19.00%		1.57	24.27%		1.70	25.34%

As can be seen from Table 8, the cement content *X*_*c(2)*_ in FRS with a certain fiber content has an increasing trend with the cement content *X*_*c(0)*_ in BCS. With the cement content increase, the reduction quantity of cement content *△X*_*c*_ and its reduction margin *△X*_*c*_*/X*_*c(0)*_ decrease first and then increase. At a certain cement content of BCS, there are various choice of FRS with different fiber content at the same strength. The cement content *X*_*c(2)*_ of the FRS gradually decreases with the increase of fiber content, while the reduction quantity *△X*_*c*_ and its reduction margin *△X*_*c*_*/X*_*c(0)*_ of cement content gradually increase. Compared with the BCS, the *△X*_*c*_ of FRS with 0.2%, 0.4%, 0.6% and 0.8% fiber content decreased by 1.09, 1.26, 1.57 and 1.70 on average, and their reduction margin *△X*_*c*_*/X*_*c(0)*_ are 17.23%, 19.00%, 24.27% and 25.34%, respectively. This is because when the fiber content increases, more fibers are randomly distributed in the FRS, the effect of the adhesion, friction, interlocking and confinement between the fiber and the solid particles of VCS could be enhanced, and the palm fiber plays a more important role to the strength of the VCS.

### 4.4 The linear interpolation method to calculate the fiber content and cement content of FRS

In theory, the fiber content *W*_*f*_ and cement content *X*_*c(2)*_ of the FRS with the same strength of BCS can be calculated through Formula (4) and Formula (7). For convenience, we could look-up the values of the *W*_*f*_ and the *X*_*c(2)*_ by Tables 7 and 8, respectively. For the values that cannot be obtained from Tables 7 or 8 (for example, the *W*_*f*_ corresponding to the *X*_*c(0)*_ with a value of 4.1 in Table 7 or the *X*_*c(2)*_ corresponding to the *X*_*c(0)*_ with a value of 5.3 in Table 8), the values of *W*_*f*_ and *X*_*c(2)*_ can be calculated by linear interpolation based on the data in Tables 7 and 8.

Table 9 gives the theory value of *W*_*f*_ and the approximative value of *W’*_*f*_ obtained by Formula (4) and linear interpolation, respectively. Table 10 gives the theory value of *X*_*c(2)*_ and the approximative value of *X’*_*c(2)*_ obtained by Formula (7) and linear interpolation, respectively. The fiber ratio and cement content of the reinforced base material calculated by the interpolation method are recorded as *W’*_*f*_ and *X’*_*c(2)*_, respectively.

**Table 9:** The *W*_*f*_ and *W’*_*f*_ calculated by theoretical method and interpolation method.

**BCS of EF section**	*Xc(0)*/%	4.1	4.3	4.5	4.7	4.9	5.1
**FRS**	*Wf*	0.038	0.065	0.108	0.178	0.291	0.47
	*W’f*	0.04	0.065	0.11	0.185	0.3	0.48
margin of Error		5.0%	0.0%	1.8%	3.8%	3.0%	2.1%

**Table 10:** The *X*_*c(2)*_ and *X’*_*C(2)*_ calculated by theoretical method and interpolation method.

BCS of FB section	FRS											
*Xc(0)*/%	*W*_*f*_ = 0.2%			*W*_*f*_ = 0.4%			*W*_*f*_ = 0.4%			*W*_*f*_ = 0.8%		
	*X*_*c(2)*_	*X’*_*c(2)*_	margin of Error	*X*_*c(2)*_	*X’*_*c(2)*_	margin of Error	*X*_*c(2)*_	*X’*_*c(2)*_	Error	*X*_*c(2)*_	*X’*_*c(2)*_	margin of Error
4.9	3.70	3.64										
5.1	4.10	4.08										
5.3	4.38	4.37	0.5%	4.06	4.01	2.5%	3.63	3.03	30.0%			
5.5	4.62	4.62	0.0%	4.42	4.41	0.5%	4.17	4.14	1.5%	3.86	3.78	4.0%
5.7	4.84	4.84	0.0%	4.69	4.68	0.5%	4.48	4.47	0.5%	4.27	4.25	1.0%
5.9	5.05	5.04	0.5%	4.92	4.92	0.0%	4.73	4.72	0.5%	4.54	4.54	0.0%
6.1	5.24	5.24	0.0%	5.13	5.12	0.5%	4.94	4.94	0.0%	4.77	4.76	0.5%
6.3	5.43	5.43	0.0%	5.31	5.31	0.0%	5.13	5.12	0.5%	4.95	4.95	0.0%
6.5	5.61	5.61	0.0%	5.49	5.48	0.5%	5.29	5.29	0.0%	5.11	5.11	0.0%
6.7	5.77	5.77	0.0%	5.64	5.63	0.5%	5.44	5.43	0.5%	5.25	5.24	0.5%
6.9	5.91	5.91	0.0%	5.77	5.76	0.5%	5.56	5.55	0.5%	5.35	5.35	0.0%
7.1	6.03	6.03	0.0%	5.87	5.86	0.5%	5.65	5.64	0.5%	5.43	5.42	0.5%
7.3	6.12	6.12	0.0%	5.95	5.94	0.5%	5.71	5.71	0.0%	5.48	5.48	0.0%
7.5	6.19	6.19	0.0%	6.00	6.00	0.0%	5.76	5.76	0.0%	5.52	5.52	0.0%
7.7	6.24	6.24	0.0%	6.04	6.04	0.0%	5.79	5.79	0.0%	5.54	5.54	0.0%
7.9	6.28	6.27	0.0%	6.06	6.06	0.0%	5.81	5.81	0.0%	5.56	5.56	0.0%

It can be seen from Table 9, compared with the *W*_*f*_ with theory calculation, the errors of the *W’*_*f*_ range from 0.0% to 5.0%, which are very small. It means that the *W*_*f*_ of FRS can be calculated by the line interpolation method based on the data in Table 5 in practice.

As shown in Table 10, when the cement content *X*_*c(0)*_ of the BCS is close to the cement content *X*_*c(F)*_ at the critical point F, the error between *X*_*c(2)*_ and the *X’*_*c(2)*_ is relatively large. In this case, the cement content *X*_*c(2)*_ of the FRS shall be calculated according to the theoretical method of Formula (7); When the cement content of the BCS is greater than the critical value *X*_*c(F)*_ of the cement content, the error between *X*_*c(2)*_ and the *X’*_*c(2)*_ is small, and the cement content of the FRS also can be obtained by the line interpolation method based on the data in Table 10.

### 4.5 The deformation characteristics of the FRS under the condition of equal strength

In order to illustrate the effect of the palm fiber substitution for cement on the mechanical properties of BCS under the condition of equal strength, two samples of the BCS and the FRS were selected for comparative analysis. The cement content of the BCS sample (marked as ①) is 8%, and its ultimate strength is 548.4 kPa; the cement content of the FRS sample (marked as ②) is 6%, and its fiber content and ultimate strength are 0.8% and 586.9 kPa, respectively. Obviously, the ultimate strength of sample ② is slightly greater than that of sample ①.

As shown in Fig 4, when the cement content is constant, the ultimate strength of the FRS is positively correlated with the fiber content. Therefore, it can be inferred that the fiber content is less than 0.8% when the cement content of the ideal sample (marked as ③) of the FRS with the same strength as the control sample ① is 6%. Studies have shown that when the cement content of the BCS keeps constant, the higher the fiber content is, the better the ductility and the ability to resist deformation and failure of the VCS are (Huang et al 2021). That is, the ability to resist deformation and failure of the ideal sample ③ is better than that of the sample ②.

**Fig 7:**
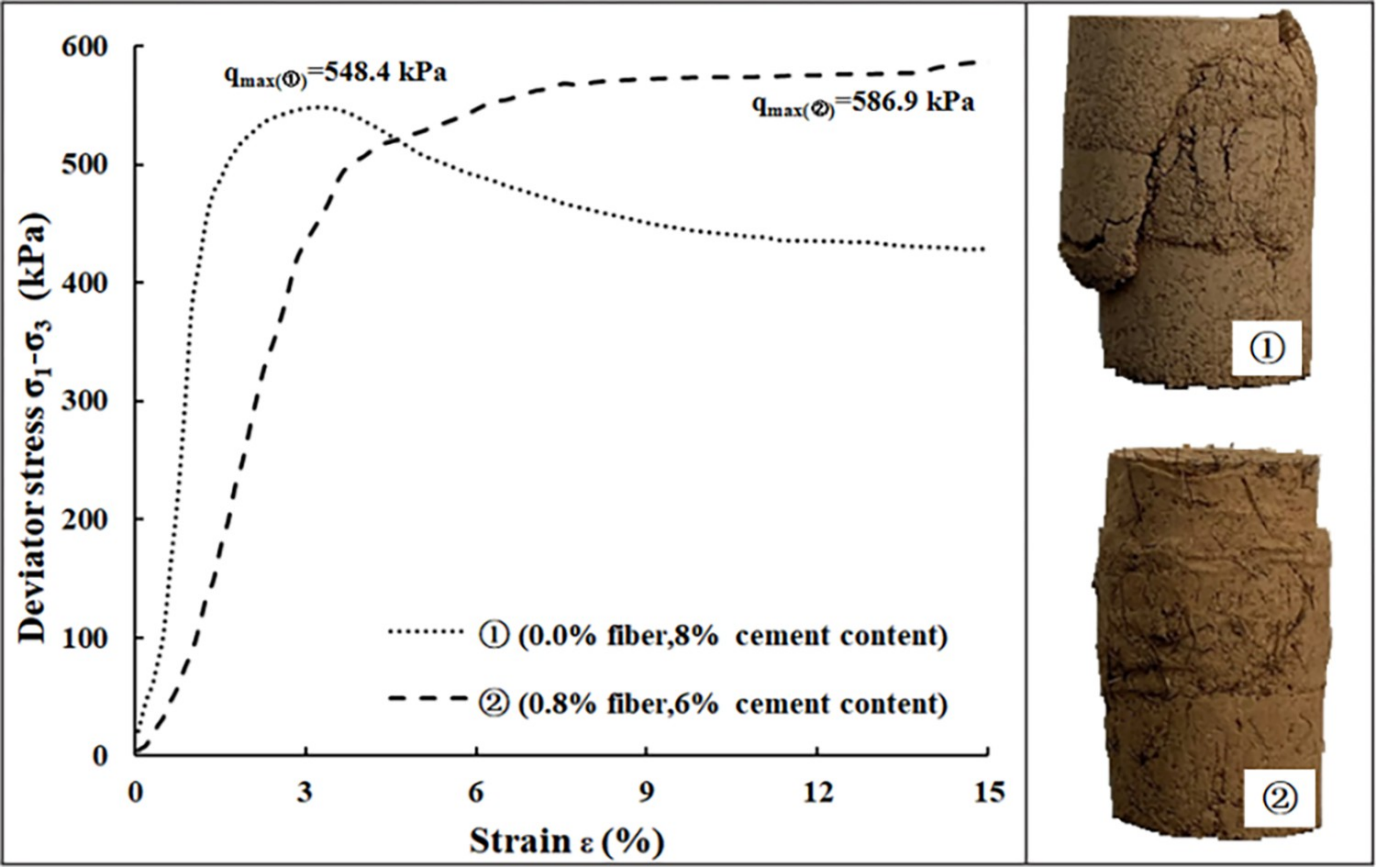
Stress-strain curves and failure photos of BCS sample and FRS sample.

Fig 7 shows the stress-strain curves of specimens ① and ② and their failure photos.

As can be observed, the sample ① possesses a softening stress-strain curve, and when its axial strain reaches up to 3%, the sample is damaged. However, the curve of the sample ② is characterized as strain hardening feature, and when the axial strain reaches up to 3%, it enters the stage of plastic deformation. With the further increasing of axial strain (>5%), the strain hardening feature of the sample ② appears gradually, indicating a better ductility of FRS.

Apart from the stress-strain curve, the number of cracks on the surface of the sample ② is obviously less than that of the sample ①. And the length of its cracks are also shorter than those of the sample ①, which means that the sample ② has better integrity and structure. Overall, with the comparation of the sample ① and sample ②, it can be concluded that the FRS has better deformation characteristics than the BCS under the condition of equal strength.

In summary, the ability of the BCS sample (①), the FRS sample (②) and the ideal FRS sample (③) to resist deformation and failure from large to small is ③>②>①. This shows that the addition of palm fiber in VCS helped in improving the ability to resist deformation and failure under the condition of equal strength.

## 5 Conclusion

This paper conducted unconsolidated undrained triaxial shear tests on blank and reinforced VCS under different cement content and fiber ratio factors, and the following main conclusions were obtained.

1. The reinforcement of plam fiber can obviously improve the mechanical properties of VCS. The softening characteristics of the stress-strain curve of the reinforced substrate gradually weaken with the increase of fiber content. The eccentric stress and peak strength of reinforced substrate are higher than those of unreinforced substrate, and increase with the increase of fiber content under the same axial strain variation.
2. The relationship between the peak strength and cement content of VCS can better fitted by the Boltzmann function. There is a critical point *Xc(F)* when fiber replaces cement under the condition of equal strength. Cement can be completely replaced with plam fiber when the cement content of unreinforced substrate was below *Xc(F)*.While the cement content of unreinforced substrate is higher than *Xc(F)*, cement can be partially reduced through the reinforcement compensation of plam fiber. Under the condition of equal strength, the average decrease of cement content with the increase of fiber content from 0.2% to 0.8%is 17.23%, 19.00%, 24.27% and 25.34%, respectively.
3. The cement content of reinforced substrates can be determined by approximate calculation using an equal strength model or interpolation method in engineering practice.
4. Fibers can not only compensate for the strength loss of the substrate caused by a decrease in cement content, but also improve the ductility of the substrate and enhance its ability to resist deformation and damage.
